# ECG challenge: a patient with recurrent syncope preceded by severe lumbar back pain

**DOI:** 10.1093/ehjcr/ytae138

**Published:** 2024-03-19

**Authors:** Luke Byrne, Caleb Powell, Gerard Fahy

**Affiliations:** Department of Cardiology, Cork University Hospital, Wilton, Co. Cork T12DC4A, Ireland; Department of Cardiology, Cork University Hospital, Wilton, Co. Cork T12DC4A, Ireland; Department of Cardiology, Cork University Hospital, Wilton, Co. Cork T12DC4A, Ireland

A 72-year-old lady presented following three syncopal episodes each lasting 15 s with prompt recovery without other cardiac symptoms. She experienced severe lumbar pain, peaking in intensity the night before admission, which was triggered by lifting a heavy flower pot three weeks prior. Clinical examination was normal with no features of decompensated heart failure. She had a family history of long QT syndrome (LQTS) 1 and carried a KCNQ1 mutation. Serial QT interval assessments prior to presentation, including during Holter monitoring and exercise testing, were reassuring. She had a history of persistent atrial fibrillation requiring electrical cardioversion one year prior. Sinus rhythm was maintained on flecainide 75 mg b.i.d. and nebivolol 5 mg o.d. following inpatient initiation with no effect on the QT interval. Her other medications included rivaroxaban 20 mg, atorvastatin 40 mg, and levothyroxine 50 μg. Biochemistry was normal apart from troponin that was elevated at 60 ng/L (normal < 16 ng/L). Frequent runs of non-sustained polymorphic ventricular tachycardia (PMVT) on cardiac monitoring were observed. Below is her 12-lead ECG on presentation.

**Figure:**
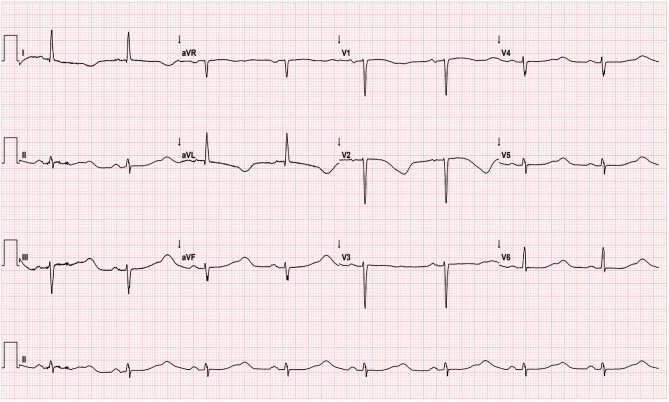


Which of the following best describes the pertinent findings from the above ECG?

- Anterolateral t wave inversion and QT prolongation due to myocardial ischaemia
- QT prolongation with evidence of macroscopic T wave alternans (TWA)
- ECG findings are suggestive of an underlying dilated cardiomyopathy (DCM)
- QT prolongation due to flecainide
- Left ventricular hypertrophy (LVH) with ‘strain’ pattern

Answer = (b).

## Discussion

The ECG shows QT prolongation with subtle macroscopic TWA. This is appreciated in the rhythm strip and is observed between beats 2 and 3, and subsequently 4 and 5. T wave alternans refers to the beat to beat variation in the timing, morphology or amplitude of the T wave and suggests instability of myocardial repolarization. It’s predictive of malignant ventricular arrhythmias (VA). Her history is not suggestive of myocardial ischaemia, and this degree of QT prolongation is not usually seen in DCM alone. Flecainide may prolong the QT interval by widening the QRS. Finally, the ECG is not in keeping with LVH.

Based on the clinical information and ECG findings, what is the next most appropriate step to confirm the diagnosis?

- Perform a coronary angiogram to out rule obstructive coronary artery disease
- Plasma metanephrines
- Perform a 24 h Holter monitor to characterize mean QT intervals
- Transthoracic echocardiogram (TTE)
- Check serial troponin levels to determine to need for coronary angiogram

Answer = (d).

Transthoracic echocardiogram is a first line diagnostic test in patients who present with ECG abnormalities and elevated troponin. The TTE revealed LVEF of 25–30% with basal to mid LV akinesia and preserved apical contraction, suggestive of atypical takotsubo cardiomyopathy (TTS). QTc prolongation and malignant VA are seen in TTS in 3–8.6% of cases.^1^ Flecainide was discontinued. LV function normalized 9 days later, and coronary angiography was normal. TTS may be preceded by an emotional, physical, or combined trigger in the majority, but not all cases. Therefore, TTS in the context of LQTS 1 and QT prolongation complicated by PMVT is the most likely diagnosis.

What are the most appropriate long-term management steps in this case?

- Change nebivolol to propranolol and prophylactic implantable cardioverter-defibrillator (ICD) implantation is indicated
- Change nebivolol to propranolol, add valsartan. An ICD is not indicated
- Change nebivolol to propranolol, add valsartan, and consider prophylactic ICD implantation
- Continue nebivolol and consider prophylactic ICD implantation
- Continue nebivolol, add valsartan, and consider prophylactic ICD implantation

Answer = (c).

Propanolol or nadolol is recommended as first line pharmacotherapy of LQTS 1. The addition of valsartan may reduce further episodes of TTS and improve cardiac remodelling. The 2022 ESC guidelines on the management of ventricular arrhythmias do not offer specific recommendations in patients with LQTS 1 and TTS as this combination is rare.^2,3^ Our patient’s QTc interval shortened as TTS resolved and returned to baseline on follow-up. TTS recurrence is not uncommon and after discussion with the patient, she opted for ICD implantation, due to the unpredictable risk of future arrhythmia, especially in the short-term.

**Consent:** Informed consent was taken from the patient for the purposes of this article.

**Funding:** No funding was obtained for the purposes of this research.

## Data Availability

Data from this case report are available on request. Please contact the corresponding author.
